# Human Immune Responses and the Natural History of *Neisseria gonorrhoeae* Infection

**DOI:** 10.3389/fimmu.2018.03187

**Published:** 2019-02-19

**Authors:** Angela Lovett, Joseph A. Duncan

**Affiliations:** ^1^Department of Pharmacology, University of North Carolina at Chapel Hill, Chapel Hill, NC, United States; ^2^Division of Infectious Diseases, Department of Medicine, University of North Carolina at Chapel Hill, Chapel Hill, NC, United States

**Keywords:** *Neisseria gonorrhoeae*, immune response, natural history, human infection, immunoglobulin, lymphocyte

## Abstract

The intimate relationship between humans and *Neisseria gonorrhoeae* infections span centuries, which is evidenced in case reports from studies dating back to the late 1700s and historical references that predate medical literature. *N. gonorrhoeae* is an exclusive human pathogen that infects the genital tract of both men and women as well as other mucosal surfaces including the oropharynx and rectum. In symptomatic infections, *N. gonorrhoeae* induces a robust inflammatory response at the site of infection. However, infections can also present asymptomatically complicating efforts to reduce transmission. *N. gonorrhoeae* infections have been effectively treated with antibiotics since their use was introduced in humans. Despite the existence of effective antibiotic treatments, *N. gonorrhoeae* remains one of the most common sexually transmitted pathogens and antibiotic resistant strains have arisen that limit treatment options. Development of a vaccine to prevent infection is considered a critical element of controlling this pathogen. The efforts to generate an effective gonococcal vaccine is limited by our poor understanding of the natural immunologic responses to infection. It is largely accepted that natural protective immunity to *N. gonorrhoeae* infections in humans does not occur or is very rare. Previous studies of the natural history of infection as well as some of the humoral and cellular immune responses to infection offer a window into the issues surrounding *N. gonorrhoeae* vaccine development. In this review, we summarize the current body of knowledge pertaining to human immune responses to gonococcal infections and the role of these responses in mediating protection from *N. gonorrhoeae*.

## Introduction

*Neisseria gonorrhoeae* is a bacterial sexually transmitted pathogen that most commonly infects the lower genital tract, the cervix in women, and anterior urethra in men. *N. gonorrhoeae* can also infect other mucosal surfaces, particularly the pharynx and rectum. Symptoms associated with disease including purulent urethral or cervical discharge and discomfort at the site of infection are due to the pathogens ability to induce robust localized inflammation within the host. However, asymptomatic infections with *N. gonorrhoeae* are common and may serve as a significant reservoir of transmissible bacteria in the population. In women, untreated infections can ascend to the upper genital tract leading to a number of health complications including pelvic inflammatory disease and infertility. Rarely, infection with *N. gonorrhoeae* disseminates leading to septic arthritis and skin manifestations. Antibiotic resistance in *N. gonorrhoeae* strains is on the rise worldwide and effective treatment options have become limited. Although individuals with gonococcal infections are known to produce anti-gonococcal humoral immune responses, it is clear that most of these responses are insufficient for providing protection from future infection. We will review the current state of knowledge regarding immune responses to gonococcal infections and the role of those responses in mediating protection from *N. gonorrhoeae* in humans. Better understanding of the immune response to natural infection with *N. gonorrhoeae* is vital for the prevention of disease transmission and the development of an effective gonococcal vaccine.

## Natural History of *Neisseria gonorrhoeae* Infection

Our understanding of the natural history of *N. gonorrhoeae* infection is hampered by a lack of rigorous scientific studies of microbiologically defined *N. gonorrhoeae* infection from the pre-antibiotic era. Since the introduction of sulfa-based antibiotics and subsequently penicillin, antibiotic treatment for men with symptomatic *N. gonorrhoeae* infection, usually urethritis, has been the standard of care. In studies of men seeking care for gonococcal urethritis subjects reported average incubation periods of ~6–8 days between presumed exposure and onset of symptoms (1, 2). However, some individuals reported symptom onset as early as 1–2 days. These studies also indicated that men with symptomatic gonococcal urethritis were symptomatic on average for 7 days before seeking care, though that time ranged from 1 day to 1 year. Because current guidelines indicate men with symptomatic gonorrhea should be treated with antibiotics, there are no prospective studies describing natural clearance or natural progression of symptomatic infection. However, some information about the persistence of symptomatic *N. gonorrhoeae* infection can be drawn from treatment failures in therapeutic trials of antibiotics for gonococcal urethritis. Svinland et al. examined bacterial clearance after treatment with flumequine in 239 patients with uncomplicated gonorrhea (3). Although multiple dosing regimens with flumequine were effective at curing the vast majority of patients, there was a small number of patients who failed to clear the infection following treatment. *N. gonorrhoeae* infection was found to persist at the test of cure obtained after 14 days in 10 subjects. Six of those subjects harbored strains with high level flumequine resistance. Those six strains represented all high-level resistant strains found in the study and therefore represented a complete cohort of subjects who received ineffective antibiotic therapy in the study. The persistence of infection in all of these subjects suggests that a large proportion of symptomatic *N. gonorrhoeae* infections that go without treatment are likely to persist at least 14 days. Treatment failures have also been reported in a number of other therapeutic trials that also support the hypothesis that *N. gonorrhoeae* can infect the lower genital tract and persist in the face of localized inflammatory response for at least 14 days (4, 5). To characterize the average bacterial load during infection, Isbey et al. analyzed the urine and semen of men with symptomatic urethritis.

Since the treatment of men with asymptomatic gonorrhea has not always been the standard for clinical management of *N. gonorrhoeae* infection, Handsfield and colleagues conducted a prospective study of the natural history of asymptomatic male infection in the early 1970's. Asymptomatic men were identified via positive *N. gonorrhoeae* urethral cultures from men requesting STI screening or men that were contacts of women positive for symptomatic gonorrhea at Seattle STD clinics. Of the 28 patients examined weekly, 18 remained asymptomatic until they were treated, which varied from 7 to 165 days. Of the remaining 10 subjects, 5 developed urethritis, and the other 5 spontaneously cleared the infection (6). The determinants of symptomatic vs. asymptomatic infection remain unknown. The total number of *N. gonorrhoeae* recovered per urine sample (~6^*^10^6^ CFUs) and from semen (~7^*^10^6^ CFUs) suggests that *N. gonorrhoeae* are carried and/or excreted at large quantities during male infection (7). Experimental gonococcal infection with male volunteers mimics much of the clinical features of naturally acquired infections and has provided some insight on the natural history of early symptomatic infection. Infected subjects often develop dysuria and urethritis with the onset of symptoms ranging from ~1 to 6 days post inoculation and *N. gonorrhoeae* can be recovered from the urine in as little as 2 h following inoculation. The quantity of bacteria recovered (~10^2^-10^5^ CFUs/sample) does not appear to correlate with the severity of infection symptoms (8). Overall, these studies highlight the ability of *N. gonorrhoeae* to persist for prolonged periods in both symptomatic and asymptomatic men. Additionally, it is clear that asymptomatic infection can progress to symptomatic infection across a broad time spectrum. The determination of the natural rate of clearance of infection is complicated by the need to treat infected individuals with effective antibiotics.

Studies of the natural history of *N. gonorrhoeae* infection in women are also limited due to a standard of care that requires the use of antibiotics to treat known *N. gonorrhoeae* infection. In a case survey comparing cure rates of *N. gonorrhoeae* infection after treatment with sulfathiazole or penicillin, patients were followed for 3 months before being declared cured. The majority of the observed patients were female in this report. Sulfathiazole treatment had a 21% failure rate, with some patients found to have positive cultures 3 months or longer after initial therapy (9). A study of women determined to be recently exposed (average of 11 days after exposure) to *N. gonorrhoeae* provided some insight into the acquisition and presentation of *N. gonorrhoeae* infection. Twenty-six women were identified through contact tracing of partners of men with gonococcal urethritis. Of the 26 subjects, 19 were found to be infected with *N. gonorrhoeae*. Risk of infection after exposure in these women increased with number of exposures to the infection (sexual encounters with the infected contact): 6/12 women with one exposure were found to be infected while 6/7 women with two exposures and 7/7 women with more than two exposures were found to be infected. Of the 19 infected subjects, 9 subjects had clinically defined pelvic inflammatory disease or tenderness of the adnexa suggestive of inflammation in the upper reproductive tract (10). Because of the risk for ascending infection, study of the natural history of asymptomatic infection in women, once identified is not considered ethically acceptable. However, Stupiansky et al. performed a study using self-collected vaginal swabs samples collected in a prospective cohort designed to study sexual health in adolescent women. In this study, subjects underwent examination and clinic-based STD screening every 3 months, 4 times more frequently than recommended for asymptomatic adolescent women. In between screenings, the subjects self-collected cervicovaginal swabs and kept diaries of sexual activity and urogenital symptoms. *N. gonorrhoeae* DNA was identified in cervicovaginal samples collected prior to the identification of *N. gonorrhoeae* infection at a quarterly visit in 18 women. Although the quantity of *N. gonorrhoeae* DNA found in self-collected swabs from individual women varied greatly, the mean bacterial load (~10^3^-10^5^ CFUs/ sample) was similar regardless of the length of time of infection. Additionally, women with *Chlamydia trachomatis* coinfections displayed higher mean bacterial load, though the difference in *N. gonorrhoeae* DNA levels between *C. trachomatis* co-infected and uninfected subjects was not statistically significant. Because the longest period between clinic-based STD screens in this was 12 weeks, persistent infection longer than 12 weeks could not be identified in this group. Interestingly, vaginal discharge and dysuria were reported in the diaries of 3 of the 18 women. However, the presence of symptoms did not correlate with bacterial load (11). These studies suggest that both symptomatic and asymptomatic *N. gonorrhoeae* infections can persist in women for at least 12 weeks. Further, the frequency of natural clearance in the setting of prolonged infection was not reported for these 18 subjects, but it at least some infections persisted from initial onset up to the quarterly in person visit. Taken together with evidence of persistent symptomatic and asymptomatic infections in men, this report suggests that *N. gonorrhoeae* is capable of evading or resisting host immune responses to infection.

**Figure 1:**
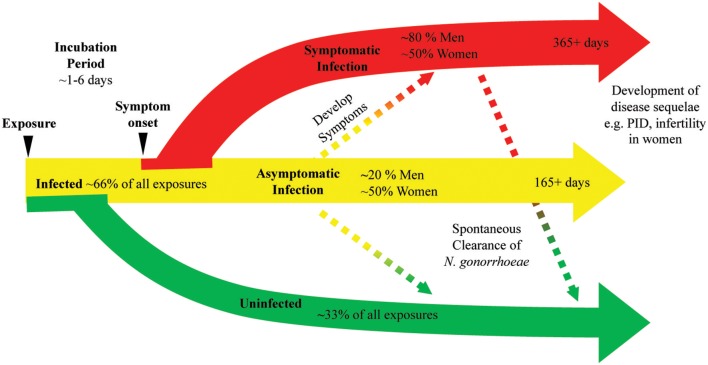
*N. gonorrhoeae* exposure can lead to bacterial clearance or prolonged infection with or without symptoms. A diagram of the natural history of *N. gonorrhoeae* infection after exposure demonstrates as many as 33% of exposed individuals will not develop infection. Infected individuals can have symptoms or remain asymptomatic. Asymptomatic infection can eventually progress to symptomatic infection, with studies indicating this progression may occur in as many as 25% of asymptomatic infections. Symptomatic infection can persist at least 14 days and infection is documented to last as long as 1 year. Asymptomatic infection is documented to persist as long as 165 days with as many as 25% of asymptomatic infections clearing over that time frame. The use of antibiotic therapy in all studies generating these data limit our knowledge of the actual rates of bacterial clearance and length of time chronic infection can persist.

Historical writings like those from James Boswell, an eighteenth century biographer who reported 20 episodes of symptoms consistent with gonococcal infection, have long been offered as anecdotal evidence that *N. gonorrhoeae* infection does not induce protective immunity (12). Studies that provide insight into the natural history of *N. gonorrhoeae* infection suggest that prior infection with *N. gonorrhoeae* induces little protective immune responses to the pathogen. Platt's study of *N. gonorrhoeae* acquisition by women exposed to men with gonococcal urethritis demonstrated that 1–7 women exposed and uninfected had prior history of *N. gonorrhoeae* infection while 10 of 19 exposed and infected individuals had prior history of *N. gonorrhoeae*, which is consistent with prior infection not leading to protective immune response (10). An epidemiologic study of *N. gonorrhoeae* infections in rural North Carolina found 14.8% of *N. gonorrhoeae* infected individuals experienced a second infection during the study period. The *N. gonorrhoeae* strain recovered from those repeatedly infected individuals was more likely to be the same serovar as the strain recovered from their initial infection than an alternative serovar, suggesting that strain specific immune responses were inadequate to provide protection from *N. gonorrhoeae* (13). In the prospective cohort study of adolescent women that described the presence of *N. gonorrhoeae* DNA in self-collected cervicovaginal swabs, no difference in the quantity of *N. gonorrhoeae* DNA was observed between individuals with a history of prior infection (11). Overall, the data from clinical studies of *N. gonorrhoeae* infection suggest that immune clearance of infection and protection from repeated infection are largely inadequate leading to prolonged symptomatic and asymptomatic infections (Figure 1).

## Humoral Immune Responses to *N. gonorrhoeae* Infection (Table 1)

Individuals infected with *N. gonorrhoeae* have been shown to produce anti-gonococcal antibodies in sera, seminal plasma, and cervical secretions. Although increases in the different immunoglobulin classes are detected in the serum and secretions of infected individuals, the difference between infected and uninfected individuals have often been reported as modest. Hedges et al. tested both serum and secretions of infected and uninfected male and female attendees of an STD clinic for antibodies against the MS11 strain and homologous infecting strains. When compared to uninfected patients, slight increases in serum IgA1 from female patients and serum IgG from male patients against the MS11 strain were observed. The level of serum IgA and IgG against infecting isolates did not drastically change over the 6-week observation period. Additionally, no difference in anti-gonococcal antibody levels was noted between individuals with no prior history of infection and previously infected individuals regardless of their current infection status (20). However, this study focused on antibody directed against fixed whole bacteria and not specific antigens, suggesting that the bulk of the reactive antibodies measured in this assay resulted from cross reactive immunoglobulins directed against commensal bacterial targets or that the assay was performed in a way that detected high level non-specific antibody binding that masked the ability to detect specific immune responses to gonococcal infection. Antigen specific antibody responses have been reported for a number of gonococcal polysaccharide and protein antigens, in some studies preadsorption of sera with other bacterial species may have contributed to the ability to detect antibodies that developed specifically during *N. gonorrhoeae* infection.

**Table 1:** Anti-gonococcal immunoglobulins identified in humans.

**Antigen**	**LOS^a^^,^^b^^,^^c^**	**Pili^a^^,^^d^**	**Protein I (PorB)^a^**	**Protein II (Opa)^a^**	**Protein III (Rmp)^a^**	**Lip^a^**	**Tbp^f^**	**Whole Bacteria^g^**
Pre-existing Antibody (or present in healthy controls)	+	+	+	+	+	+	+	+
Post-infection Antibody	++	++	+	+	+	+	+	+
Immunoblot/ELISA	I/E	E	I	I	I	I	E	E

a Hicks et al. (14);

b Schmidt et al. (15);

c Yamasaki et al. (16);

d Meittenen et al. (17);

e Plummer et al. (18);

f Price et al. (19);

g *Hedges et al. (20)*.

Polysaccharides are a major source of microbial antigens from pathogenic Neisseria species, as well as many other bacteria. Unlike *Neisseria meningitidis, N. gonorrhoeae* does not produce a polysaccharide capsule, which is a major immunogenic, saccharide-based antigen for *N. meningitidis*. However, both *N. gonorrhoeae* and *N. meningitidis* produce lipooligosaccharide (LOS) which makes up roughly 50% of the mass of the outer membrane of the bacteria. LOS from *N. gonorrhoeae* and *N. meningitidis* share a common core oligosaccharide along with lipid A structure (21). The structure of the oligosaccharide of LOS is determined by the expression of multiple phase variable saccharide transferases (22). Thus, even within a single strain there can be substantial LOS heterogeneity. The ability of these variations in structure to lead to heterogeneity in antigenic epitopes that can be recognized by antibodies was demonstrated clearly by the characterization of *N. gonorrhoeae* LOS from 20 different strains with a panel of murine monoclonal antibodies (23). Despite these variations in LOS structure, almost all *N. gonorrhoeae* strains examined by Gulati and colleagues maintain expression of the LOS structure recognized by monoclonal antibody 2C7 (24). Antibodies to LOS have been detected in both normal human sera and in sera from *N. gonorrhoeae* infected individuals. Sera collected from a small subset of male subjects in a gonococcal pilus-based vaccine trial were used to characterize antigen specific antibody responses during acute gonococcal infection. These subjects who received the placebo vaccination, reported no previous history of STIs, had a negative culture at the start of the trial and developed gonococcal urethritis during the trial period. Sera were collected bi-weekly over the course of 8 weeks. This study found 4 of 13 subjects carried pre-existing LOS antibodies that presumably arose from cross reactivity with polysaccharides from other commensal bacteria species. Of the remaining 9 subjects who did not have preexisting antibodies that recognize *N. gonorrhoeae* LOS, 6 developed LOS antibodies after developing *N. gonorrhoeae* infection (14). In another study, sera collected from patients with complicated gonococcal infections [8 disseminated gonococcal infections, 4 cases of pelvic inflammatory disease (PID), and 1 case of epididymitis] was used to characterize antibody responses to *N. gonorrhoeae* outer membrane proteins. Sera obtained from a majority of patients (9/13) exhibited antibody responses to LOS (4). Antibodies against LOS were also studied by researchers using an experimental human male urethral inoculation to test whether recent gonococcal urethritis led to protection from reinfection. Some immunoreactivity toward *N. gonorrhoeae* LOS was detected in serum from 21 of 24 subjects prior to inoculation with *N. gonorrhoeae* for the first time. Of the 14 subjects in the study who were rechallenged, 6 of 14 demonstrated an increase in anti-LOS antibody demonstrated by immunoblot, and 9 of 14 had at least a 2-fold increase in antibody titer measured by ELISA (15). Consistent with baseline observations of subjects in these studies, the presence of *N. gonorrhoeae* LOS-directed antibodies was detected from pooled sera taken from eight volunteers with no history of gonococcal infection (16). The predominant antibodies that bound LOS in this study were of the IgG class. Affinity purified anti-oligosaccharide IgG isolated from normal human serum was found to contain bactericidal activity toward a serum sensitive strain of *N. gonorrhoeae*. In a separate study, Yamasaki and colleagues found that *N. gonorrhoeae* LOS-binding IgG2 from NHS recognized at least 3 different oligosaccharide (25). Thus, numerous studies indicate that the majority, but not all, humans mount antibody responses to a variety of saccharide epitopes found in the lipooligosaccharide of *N. gonorrhoeae* in response to mucosal or invasive infection with the bacteria.

A number of studies have surveyed whether different gonococcal protein antigens induce humoral immune responses during infection. Sera isolated from 68 patients with uncomplicated and 35 women with PID were used to characterize antibody responses to gonococcal pili (17). The mean antibody levels of IgG, IgA, and IgM against *N. gonorrhoeae* pilus was significantly higher in men with urethritis (~2-fold increase) and women with cervicitis (~4-fold increase) when compared to sera from uninfected patients. Anti-pilus antibodies were also significantly higher in sera from women with confirmed gonococcal PID when compared to sera from patients with non-gonococcal PID. In addition to testing for anti-LOS antibodies, sera isolated from male volunteers participating in pilus vaccine trial who acquired gonorrhea and who reported no previous history of gonococcal infection were analyzed for the presence on anti-gonococcal protein antibodies using immunoblot analysis. Pre-existing antibodies were detected in 12 of 13 subjects by western blot analysis using pre-infection serum. Not only were preexisting antibodies against LOS detected but antibodies against a number of abundant outer-membrane proteins were observed including: Protein I (PorB), Protein III (Rmp), Pili, and Lip. The authors suggested that the preexisting antibodies to LOS and *N. gonorrhoeae* proteins may be a result of cross reactivity to antigens from nasal carriage of *N. meningitidis*. After acquisition of *N. gonorrhoeae* infection, 9 of 13 subjects developed IgG serum antibody responses with antibodies against all 6 major outer membrane antigens analyzed in this study (LOS as well as Proteins I, II, III, Pili, and Lip). In the aforementioned study by Hook et al. in which sera collected from 8 patients with complicated *N. gonorrhoeae* infections, Protein I antibodies were found in sera collected from all 13 patients with complicated gonococcal infections (4). Antibodies against both PorB (Protein I) and Opa (Protein II) were also detected in the sera of almost all commercial sex workers in Nairobi, Kenya, that were enrolled in a prospective cohort study of sexually transmitted infections (18, 26). The combination of increased levels of gonococcal antigen-directed antibodies in infected humans combined with prevalent antibodies in a highly exposed cohort, suggest that infection with *N. gonorrhoeae* can cause the development of antibodies directed toward the pathogen. A study of antibody titer or development of specific pathogen-directed antibodies during prolonged infection or after resolution of infection would be required to demonstrate the antibodies develop in response to infection.

Immune responses to gonococcal transferrin binding proteins were analyzed by ELISA using sera and secretions collected from patients attending STD clinics who had confirmed positive *N. gonorrhoeae* cultures. Sera from healthy volunteers were used as controls. Antibodies to both transferrin binding proteins, TbpA and TbpB, were detected in sera from men and women. However, only IgA and IgM concentrations against TbpB in women was significantly higher than the levels of anti-TBP antibodies in uninfected control sera. One male subject's serum antibody levels to TbpA and TbpB were followed for 6 months. A slight increase in IgG, IgM, and IgA was observed 1 month after infection and the levels returned to baseline for the following 5 months of the study, suggesting that the antibody titers increased in response to infection (19). In the previously mentioned two phase study utilizing urethral challenge and rechallenge, with *N. gonorrhoeae* in male volunteers with no previous history of gonococcal infection, pre-infection sera from each volunteer contained IgG to at least one of the major gonococcal Outer Membrane Proteins. A majority (18/24) of the volunteers had pre-existing anti-pilus antibodies. Immunoblots conducted with pre- and post-infection sera were reported from a subset of these subjects. Increases in sera antibodies to outer membrane proteins, particularly IgG, was detected following infection in most subjects however the pattern of recognized proteins varied from subject to subject. Though *N. gonorrhoeae* exposure or infection prior to enrollment was thought to explain the preexisting detectable antibodies to *N. gonorrhoeae* antigens in the Kenyan commercial sex-worker cohort study, the number of different serogroups of PorB recognized by sera from subjects in this cohort rose with the number of study visits, suggesting that ongoing exposure to new strains of *N. gonorrhoeae* was resulting in additional antibody responses to that gonococcal antigen (26). Overall, these studies also support the finding that as with lipooligosaccharide, pre-existing antibodies to gonococcal protein antigens are common in humans but antibodies to some antigens do appear to increase in response to infection.

While there is clear *in vitro* evidence that some antibodies directed toward *N. gonorrhoeae* antigens can promote either complement mediated killing or opsonophagocytic killing of the bacteria, whether these antibodies can prevent human infection or play a role in immunologic clearance of the pathogen remains an open question. In a study of experimental human infection followed by rechallenge with *N. gonorrhoeae*, 7 of 8 subjects who experienced at least a 2-fold increase in anti-LOS antibody resisted reinfection while only 1 of the 6 subjects who had less than a 2-fold increase in anti LOS were susceptible to reinfection (15). Many human IgG and mouse monoclonal antibodies that recognize *N. gonorrhoeae* LOS epitopes are bactericidal (16, 27). Passive immunization with an antibody directed against the common 2c7 epitopes of *N. gonorrhoeae* is protective in a mouse model of vaginal *N. gonorrhoeae* infection (28). In a prospective study of a cohort of commercial sex workers in Kenya, a strong association between the number of different serogroups of *N. gonorrhoeae* Opa protein (protein II) recognized by antibodies in subject sera and reduced relative risk of gonococcal salpingitis was observed (18). However, in the same cohort, detectable antibody to Rmp (protein III) was associated with increased risk of gonococcal infection during the study as well as increased rate of gonococcal salpingitis (26). Although antibodies against Rmp are detected during infection, there is substantial evidence indicating that many of the antibodies against *N. gonorrhoeae* Rmp can block bactericidal activity of other *N. gonorrhoeae*-directed antibodies. Serum isolated from patients with disseminated gonococcal infection was shown interfere with *in vitro* killing of *N. gonorrhoeae* by normal human sera. Early studies aimed at identifying the mediators responsible for this blocking of normal human sera bactericidal activity demonstrated that IgG was the major source of blocking activity in sera from subjects with disseminated gonococcal infection (29, 30). IgG reactive with gonococcal outer membrane proteins was more effective at inhibiting bactericidal activity when compared to normal IgG and depletion of Outer Membrane Protein-binding IgG restored the bactericidal activity of serum. Further examination into specific antigens that elicit blocking antibody responses found that RMP-binding IgG contributed to the majority of the blocking activity observed *in vitro* (31). Price and colleagues were able to demonstrate that by preincubating *N. gonorrhoeae* with Rmp-directed IgG from immune sera decreased the binding of bactericidal antibodies from normal human sera to *N. gonorrhoeae* and inhibited killing of the bacteria by normal human sera (31, 32). Some mouse monoclonal antibodies against *N. gonorrhoeae* Rmp generated through immunization have been found to have bactericidal activity while others have been shown to have blocking activity similar to that of human sera containing anti-Rmp IgG. Analysis of two Rmp-directed monoclonal antibodies with blocking activity revealed they ound an overlapping linear epitope of the protein between amino acids 24–33, an epitope with substantial homology to an outermembrane protein from *Escherichia coli*, OmpA (33–35). Another study examining the blocking epitope of Rmp bound by antibodies in sera from humans with disseminated gonococcal infection demonstrated that antibodies directed to residues in disulfide-containing loop near the OmpA homologous region (amino acids 47–64) also had blocking activity (36). In a recent study, Gulati et al. demonstrated that passive immunization with an anti-Rmp monoclonal antibody abrogated the protective effects of the 2c7 monoclonal antibody administration in a female mouse *N. gonorrhoeae* infection model, causing both increased bacterial burden and duration of infection (28, 37). Although the Rmp protein is conserved and immunogenic, the production of anti-Rmp antibodies can inhibit the development of protective humoral responses. Overall, despite clear evidence that humans develop anti-gonococcal antibodies in response to infection, repeat infections with *N. gonorrhoeae* are common. These data indicate that the humoral immune responses induced by *N. gonorrhoeae* infection are complex and in most cases are insufficient to provide protection from infection.

## Cell Mediated Immune Responses to *N. gonorrhoeae* (Table 2)

In addition to humoral immune responses to *N. gonorrhoeae*, there is also evidence that humans with *N. gonorrhoeae* develop adaptive cellular immune responses specific to *N. gonorrhoeae* antigens during infection. Early studies of *N. gonorrhoeae* cellular immunity were conducted by measuring proliferation in response to antigen stimulation as demonstrated by radioactive adenine incorporation into DNA in cultured primary lymphocytes. Mauss and colleagues studied proliferation of lymphocytes obtained from patients with *N. gonorrhoeae* infection as well as a set uninfected male controls in response to a variety of *N. gonorrhoeae* antigen preparations. For the best characterized antigen preparation 3 of 6 female subjects and 4/4 male subjects with *N. gonorrhoeae* infection had a positive proliferative response while 1 of 7 uninfected men had a positive response (9). Two similar but larger and better controlled studies confirmed these findings, Kraus and colleagues studied lymphocyte proliferation in response to gonococcal antigens in men with gonorrhea and control male subjects with no reported history of gonorrhea (40). Lymphocyte proliferation was higher in lymphocytes from men with two or more gonococcal infections while lymphocytes from those subjects with their initial case of gonorrhea did not exhibit antigen induced proliferation. Similarly, Wyle et al. found that 21 women and 29 men with culture proven *N. gonorrhoeae* infection were found to have significantly higher proliferative indexes in response to *N. gonorrhoeae* antigen than lymphocytes from uninfected men and women who denied prior history of *N. gonorrhoeae* infection (41). Proliferative responses observed in lymphocytes from *N. gonorrhoeae* infected individuals fell below the level of detection in most individuals within 5 weeks of treatment (43). The specific *N. gonorrhoeae* antigens that are recognized by cellular immune responses in humans are poorly studied. One study demonstrated that the abundant membrane protein PorB could provide antigenic stimulation to lymphocytes from 20 and 24 of 30 *N. gonorrhoeae* infected subjects (38). In that study, a lack of PorB-induced proliferation was reported for lymphocytes from uninfected control subjects, suggesting PorB-directed lymphocytes that might arise from cross-reactive bacterial antigens are not highly represented in humans. In a separate study, lymphocytes from 8 of 8 healthy individuals demonstrated proliferative responses to treatment with *N. gonorrhoeae* IgA protease (39). It is possible these findings result from cross-reactive lymphocyte responses in lymphocytes that recognize homologous IgA proteases from commensal oral pharyngeal bacteria or even *N. meningitidis*. Genomic analysis of 4 gonococcal strains identified 23 conserved proteins with predicted T and B cell epitopes that could serve as universal antigens (44). However, the gonococcal antigens that elicit natural immune responses during infection remains largely unstudied, or at least unreported in the medical literature, at this time.

**Table 2:** Anti-gonococcal cellular immune responses identified in humans.

**Antigen**	**Protein I (PorB)^a^**	**IgA protease^b^**	**Whole Bacteria^c^^,^^d^^,^^e^^,^^f^**
**LYMPHOCYTE PROLIFERATION**			
uninfected, pre-infected	–	+	+
infected	+	NT	++
**STIMULATED CYTOKINE RELEASE: (CELL TYPE)**			
IL-4	+ (CD4 & CD8 T cell)	+ (PBMC)	+ (PBMC)
IL-10	–	+ (PBMC)	+ (PBMC)
IFNγ	–	+ (CD4 T cell, PBMC)	+ (PBMC)
TNF-α	–	+ (PBMC)	NT

NT, not tested; CD4 or CD8 T cell, cytokine detected in T cell population by intra-cellular cytokine staining; PBMC, cytokine detected in Peripheral Blood Mononuclear Cell culture supernatant by ELISA.

a Simpson et al. (38);

b Tsirpouchtsidis et al. (39);

c Kraus et al. (40);

d Wyle et al. (41);

e Mauss (9);

f *Rarick et al. (42)*.

Lymphocytes can elicit a variety of functional responses that are aimed at clearing pathogens from the host upon recognition of pathogen-derived antigens. Differential functions of T lymphocytes are mediated by differential expression of a combination of cell surface proteins and secreted mediators (cytokines and chemokines). Lymphocytes responsible for coordinating immunologic responses known as T helper cells are positive for the CD4 antigen (CD4+) and are generally sub-classified into 4 broad groups that each have a predominant secreted cytokine: 1) Th1 which secrete interferon-gamma (IFNγ), Th2 which secrete interleukin-4 (IL-4), Th17 which secrete interleukin-17 (IL-17), and Treg which secrete interleukin-10 (IL-10). Mucosal infections typically do not induce profound differences in systemic or circulating levels of cytokines. However, sera isolated from patients with gonococcal infection were found to have modestly higher circulating levels of IL-17, IFNγ, and IL-23, when compared to sera from healthy controls (45). An inverse correlation between serum levels of IL-17 and serum levels of IFN-γ was observed suggesting that Th1 responses are blunted as Th17 responses are generated during *N. gonorrhoeae* infection. In a study of cervical immunologic factors in women undergoing STI screening at primary care clinics in Durbin, South Africa, IL-17 was found to elevated in cervical secretions of women with *N. gonorrhoeae* infection when compared to women without evidence of bacterial STI (46). IL-17 and other inflammatory cytokines (IL-1α, IL-1β, IL-12p70, TNF-α, RANTES, G-CSF, Flt3L, IL-2, IL-5, IL-15, and IL-17) were also found elevated in cervical lavage of *N. gonorrhoeae* infected women when compared to women with no detectable STI (47). In a study of women seeking care in a Nairobi STI clinic for acute abdominal pain or vaginal discharge, cervical IL-10, a cytokine produced by both regulatory T cells (Treg), and other immunoregulatory cells, was detected in 19 of 59 women with no detected bacterial STI and in 19 of 36 women with *N. gonorrhoeae* infection. Overall, published studies suggest that *N. gonorrhoeae* induces a complex lymphocyte response that is largely driven by an Il-17 or Th17 pro inflammatory response.

While there are a number of studies in which the level of cytokines in serum or site-specific fluids from *N. gonorrhoeae* infected individuals, few reported studies of the CD4+ T cell functional responses to *N. gonorrhoeae* in humans also exist. IgA protease-directed CD4 cells from healthy individuals without known *N. gonorrhoeae* infection have been shown to produce IFNγ using both elispot and intracellular cytokine staining. This finding is consistent with the ability of some gonococcal antigens to elicit Th1 type CD4 responses. In that study, cultured PBMC produced IFNγ, TNF-α, IL-4, and IL-10 in response to IgA protease antigen while only producing IFNγ and TNF-α in response to tetanus toxoid (39). This data suggests that the commensal bacteria colonization that resulted in cross reactive immune response to *N. gonorrhoeae* IgA protease elicited a polyfunctional immune response different in character from that induced by tetanus toxoid vaccination. In another *ex vivo* study of PBMC response to *N. gonorrhoeae*, cultured PBMC from 2 anonymous donors (and unknown *N. gonorrhoeae* infection history) were exposed to *N. gonorrhoeae* and CD4+ T cells were found to upregulate CD25, a T cell surface marker that is elevated in T regulatory cells and in activated CD4+ T cells. Further culture supernatant from these *N. gonorrhoeae*-treated PBMC was found to contain cytokines associated with Th1 (IFNγ), Th2 (IL-4), and Treg (IL-10) responses as well as the chemotactic factors IL-8 and MCP (42). Though the cellular source of these cytokines is unknown, the findings are consistent with the observation that *N. gonorrhoeae* infection induces a pleotropic immunologic response at the site of infection.

The role of cytotoxic T lymphocytes (CD8+ T cells), as well as cytotoxic innate immune cells like Natural Killer cells (NK cells), in gonococcal infection are unknown. Gonococcal PorB has been shown to induce robust IL-4 production in CD8+ T cells from *N. gonorrhoeae* infected individuals (38). However, contradicting effects of *N. gonorrhoeae* infection on CD8+ T cell function in humans are reported. In a longitudinal study of female commercial sex workers, Kaul and colleagues found that CD8+ T cell functional responses to HIV (in HIV infected subjects) and CMV (in HIV infected and uninfected individuals) were reduced during episodes of incident *N. gonorrhoeae* infection when compared to lymphocytes obtained when subjects were not *N. gonorrhoeae* infected (48). In contrast, a longitudinal study of HIV acquisition in a cohort of HIV negative female commercial sex workers demonstrated that early HIV-directed CD8+ T cell responses were more robust in subjects who were infected with *N. gonorrhoeae* at the time of HIV acquisition than in *N. gonorrhoeae* uninfected individuals (49). Additionally, asymptomatic anorectal *N. gonorrhoeae* infection in men who have sex with men taking pre-exposure prophylaxis to prevent HIV was found to be associated with increased activation marker in circulating CD8+ T cells (50). The adaptive immune response in cytotoxic T cell populations to *N. gonorrhoeae* infection remains largely unexplored at this time.

Cytokines from the IL-17 family are important for the recruitment of neutrophils and to induce localized antimicrobial responses. Studies in a mouse model of *N. gonorrhoeae* infections suggest that the magnitude of Th17 responses to infection are more robust than Th1/Th2 responses (51). These findings are consistent with the neutrophilic inflammatory response commonly associated with *N. gonorrhoeae* infection in humans and the inverse relationship between systemic IL-17 and IFNγ levels reported in *N. gonorrhoeae* infected individuals (45). The relative resistance of *N. gonorrhoeae* to neutrophil mediated killing may render this Th17-skewed immune response less effective at clearing the pathogen than a more robust Th1/Th2-supported immune response might, (52, 53). Further, the ability of *N. gonorrhoeae* to induce IL-10 secretion and promote or stimulate Treg cells may also reduce humoral and cellular immune responses to infection (54). Supportive of this hypothesis, manipulation of the mouse immune system to block the induction of Treg related activity or to drive more robust Th1/Th2 responses leads to enhanced clearance of *N. gonorrhoeae* in model infection. Further, stimulation of Th1 responses through intravaginal IL-12 administration during infection in the mouse vaginal infection model resulted in enhanced clearance upon rechallenge when compared to mice initially infected without supplemental IL-12 (55). Because studies of lymphocyte proliferative response demonstrated that prior natural infection was associated with significant increase in proliferative response in subjects with current *N. gonorrhoeae* infection when compared to subjects without previous infection, it appears the cell mediated immunity being measured was inadequate to provide robust protection from repeat infection. Unfortunately, there are no studies to date that measure cellular immune responses to *N. gonorrhoeae* either in naïve or infected individuals in which the subjects were subsequently prospectively followed to determine whether these cellular immune responses correlate with any degree of protection from re-infection.

Despite public health efforts, gonorrhea still remains one of the most common sexually transmitted bacterial infections responsible for ~100 million infections per year (56). The development of a gonococcal vaccine had been a focus of the field but no vaccine has been developed to the point of human trials since the failure of the *N. gonorrhoeae* pilin-based vaccine in clinical trials. The failure of the pilin vaccine was unexpected after it had shown success in preventing infection in a homologous strain challenge in humans and highlights the complex relationship between this human pathogen and its host (57). This review of published literature of human immunologic responses to *N. gonorrhoeae* largely supports the widely accepted supposition that *N. gonorrhoeae* manages to infect and re-infect humans using some combination of immunologic evasion and resistance to host mediators of clearance. However, there are some reports that support the possibility that natural clearance or protection from infection may develop in response to gonococcal infection in a small portion of individuals. The lack of a protective immune response observed in most infected humans coupled with the complex nature of antigen specific responses and limited animal infection models have all confounded past vaccine development efforts and stand in stark contrast to successful development of *N. meningitidis* vaccines. Recently, large scale efforts to develop effective vaccines toward serogroup B *N. meningitidis* which produces a poorly immunogenic capsule have led to the development of Outer membrane vesicle (OMV)-based vaccines which are effective at raising bactericidal antibodies against group B *N. meningitidis* in humans (58). Data emerging from Sexually Transmitted Infection surveillance in countries deploying OMV-based vaccine in mass vaccination campaigns suggest that the vaccine may induce some cross-species protection against *N. gonorrhoeae* infections (59–61). The mechanism by which this vaccine may be protective against *N. gonorrhoeae* is not fully understood. Further studies that evaluate the cellular and humoral responses directed against *N. gonorrhoeae* that develop after immunization with *N. meningitidis* OMV as well as comprehensive studies that combine sophisticated immunologic analysis and prospective monitoring of subjects are needed to understand both natural and the possibility of vaccine mediated immunity to *N. gonorrhoeae* infection.

## Author Contributions

AL and JD conceived and outlined this literature review and subsequently independently conducted literature searches. AL drafted initial manuscript, figures, and tables. JD edited and finalized the manuscript, figures, and tables.

### Conflict of Interest Statement

The authors declare that the research was conducted in the absence of any commercial or financial relationships that could be construed as a potential conflict of interest.

## References

[1] Schofield CB. Some factors affecting the incubation period and duration of symptoms of urethritis in men. Br J Vener Dis. (1982) 58:184–7. 10.1136/sti.58.3.184 7082980 PMC1046042

[2] Sherrard J Barlow D. Gonorrhoea in men: clinical and diagnostic aspects. Genitourin Med. (1996) 72:422–6. 10.1136/sti.72.6.422 9038638 PMC1195730

[3] Svindland HB Svarva PL Maeland JA. Quinolone derivative, flumequine, as short-term treatment for gonorrhoea. Br J Vener Dis. (1982) 58:317–20. 10.1136/sti.58.5.317 6812849 PMC1046084

[4] Hook EW III Olsen DA Buchanan TM. Analysis of the antigen specificity of the human serum immunoglobulin G immune response to complicated gonococcal infection. Infect Immun. (1984) 43: 706–9. 6198284 10.1128/iai.43.2.706-709.1984 PMC264357

[5] Tanphaichitra D Sahaphong S Srimuang S. Ofloxacin, a new quinolone in the treatment of genitourinary and enteric infections. Infection (1986) 14 (Suppl. 4):S321–3. 10.1007/BF01661308 3102387

[6] Handsfield HH Lipman TO Harnisch JP Tronca E Holmes KK. Asymptomatic gonorrhea in men. Diagnosis, natural course, prevalence and significance. N Engl J Med. (1974) 290:117–23. 10.1056/NEJM197401172900301 4202519

[7] Isbey S.F. Alcorn T. M. Davis R. H. Haizlip J. Leone P. A. Cohen M. S. (1997). Characterisation of Neisseria gonorrhoeae in semen during urethral infection in men. Genitourin Med. 73, 378–382. 9534748 10.1136/sti.73.5.378 PMC1195896

[8] Cohen MS Cannon JG Jerse AE Charniga LM Isbey SF Whicker LG. Human experimentation with *Neisseria gonorrhoeae*: rationale, methods, and implications for the biology of infection and vaccine development. J Infect Dis. (1994) 169:532–7. 10.1093/infdis/169.3.532 8158024

[9] Mauss IH. A comparison of the response of gonorrhea to sulfathiazole and penicillin; analysis of 144 cases. J Lancet (1946) 66:65–7. 21017367

[10] Platt R Rice PA Mccormack WM. Risk of acquiring gonorrhea and prevalence of abnormal adnexal findings among women recently exposed to gonorrhea. JAMA (1983) 250:3205–9. 10.1001/jama.1983.03340230057031 6417362

[11] Stupiansky NW Van Der Pol B Williams JA Weaver B Taylor SE Fortenberry JD. The natural history of incident gonococcal infection in adolescent women. Sex Transm Dis. (2011) 38:750–4. 10.1097/OLQ.0b013e31820ff9a4 21317686

[12] Rana K.J (2012). James Boswell and a History of Gonorrhoea. Available online at: https://www.theguardian.com/commentisfree/2012/jun/13/james-boswell-history-gonorrhoea (Accessed June 13, 2012).

[13] Fox KK Thomas JC Weiner DH Davis RH Sparling PF Cohen MS. Longitudinal evaluation of serovar-specific immunity to *Neisseria gonorrhoeae*. Am J Epidemiol. (1999) 149:353–8. 10.1093/oxfordjournals.aje.a009820 10025478

[14] Hicks CB Boslego JW Brandt B. Evidence of serum antibodies to *Neisseria gonorrhoeae* before gonococcal infection. J Infect Dis. (1987) 155:1276–81. 10.1093/infdis/155.6.1276 2883240

[15] Schmidt KA Schneider H Lindstrom JA Boslego JW Warren RA Van De Verg L. Experimental gonococcal urethritis and reinfection with homologous gonococci in male volunteers. Sex Transm Dis. (2001) 28:555–64. 10.1097/00007435-200110000-00001 11689753

[16] Yamasaki R Maruyama T Yabe U Asuka S. Normal human sera contain bactericidal IgG that binds to the oligosaccharide epitope expressed within lipooligosaccharides of *Neisseria gonorrhoeae*. J Biochem. (2005) 137:487–94. 10.1093/jb/mvi061 15858172

[17] Miettinen A Hakkarainen K Gronroos P Heinonen P Teisala K Aine R. Class specific antibody response to gonococcal infection. J Clin Pathol. (1989) 42:72–6. 10.1136/jcp.42.1.72 2564006 PMC1141795

[18] Plummer FA Chubb H Simonsen JN Bosire M Slaney L Nagelkerke NJ. Antibodies to opacity proteins (Opa) correlate with a reduced risk of gonococcal salpingitis. J Clin Invest. (1994) 93:1748–55. 10.1172/JCI117159 8163673 PMC294233

[19] Price GA Hobbs MM Cornelissen CN. Immunogenicity of gonococcal transferrin binding proteins during natural infections. Infect Immun. (2004) 72:277–83. 10.1128/IAI.72.1.277-283.2004 14688106 PMC343986

[20] Hedges SR Mayo MS Mestecky J Hook EW III Russell MW. Limited local and systemic antibody responses to *Neisseria gonorrhoeae* during uncomplicated genital infections. Infect Immun. (1999) 67:3937–46. 10417159 10.1128/iai.67.8.3937-3946.1999 PMC96675

[21] Mandrell RE Griffiss JM Macher BA. Lipooligosaccharides (LOS) of *Neisseria gonorrhoeae* and *Neisseria meningitidis* have components that are immunochemically similar to precursors of human blood group antigens. Carbohydrate sequence specificity of the mouse monoclonal antibodies that recognize crossreacting antigens on LOS and human erythrocytes. J Exp Med. (1988) 168:107–26. 10.1084/jem.168.1.107 2456365 PMC2188965

[22] Shafer WM Datta A Kolli VS Rahman MM Balthazar JT Martin LE. Phase variable changes in genes lgtA and lgtC within the lgtABCDE operon of *Neisseria gonorrhoeae* can modulate gonococcal susceptibility to normal human serum. J Endotoxin Res. (2002) 8:47–58. 10.1177/09680519020080010501 11981445

[23] Mandrell R Schneider H Apicella M Zollinger W Rice PA Griffiss JM. Antigenic and physical diversity of *Neisseria gonorrhoeae* lipooligosaccharides. Infect Immun. (1986) 54:63–9. 2428752 10.1128/iai.54.1.63-69.1986 PMC260117

[24] Gulati S Mcquillen DP Sharon J Rice PA. Experimental immunization with a monoclonal anti-idiotope antibody that mimics the *Neisseria gonorrhoeae* lipooligosaccharide epitope 2C7. J Infect Dis. (1996) 174:1238–48. 10.1093/infdis/174.6.1238 8940214

[25] Yamasaki R Yabe U Kataoka C Takeda U Asuka S. The oligosaccharide of gonococcal lipooligosaccharide contains several epitopes that are recognized by human antibodies. Infect Immun. (2010) 78:3247–57. 10.1128/IAI.01445-09 20479085 PMC2897362

[26] Plummer FA Chubb H Simonsen JN Bosire M Slaney L Maclean I. Antibody to Rmp (outer membrane protein 3) increases susceptibility to gonococcal infection. J Clin Invest. (1993) 91:339–43. 10.1172/JCI116190 8423230 PMC330031

[27] Apicella MA Westerink MA Morse SA Schneider H Rice PA Griffiss JM. Bactericidal antibody response of normal human serum to the lipooligosaccharide of *Neisseria gonorrhoeae*. J Infect Dis. (1986) 153:520–6. 10.1093/infdis/153.3.520 3081657

[28] Gulati S Mu X Zheng B Reed GW Ram S Rice PA. Antibody to reduction modifiable protein increases the bacterial burden and the duration of gonococcal infection in a mouse model. J Infect Dis. (2015) 212:311–5. 10.1093/infdis/jiv024 25596304 PMC4565997

[29] Mccutchan JA Katzenstein D Norquist D Chikami G Wunderlich A Braude AI. Role of blocking antibody in disseminated gonococcal infection. J Immunol. (1978) 121:1884–8. 712069

[30] Rice PA Kasper DL. Characterization of serum resistance of *Neisseria gonorrhoeae* that disseminate. Roles of blocking antibody and gonococcal outer membrane proteins. J Clin Invest. (1982) 70:157–67. 10.1172/JCI110589 6806319 PMC370238

[31] Rice PA Vayo HE Tam MR Blake MS. Immunoglobulin G antibodies directed against protein III block killing of serum-resistant *Neisseria gonorrhoeae* by immune serum. J Exp Med. (1986) 164:1735–48. 10.1084/jem.164.5.1735 3095479 PMC2188443

[32] Joiner KA Scales R Warren KA Frank MM Rice PA. Mechanism of action of blocking immunoglobulin G for *Neisseria gonorrhoeae*. J Clin Invest. (1985) 76:1765–72. 10.1172/JCI112167 3932472 PMC424204

[33] Virji M Zak K Heckels JE. Outer membrane protein III of *Neisseria gonorrhoeae*: variations in biological properties of antibodies directed against different epitopes. J Gen Microbiol. (1987) 133:3393–401. 10.1099/00221287-133-12-3393 2460579

[34] Virji M Heckels JE. Location of a blocking epitope on outer-membrane protein III of *Neisseria gonorrhoeae* by synthetic peptide analysis. J Gen Microbiol. (1989) 135:1895–9. 10.1099/00221287-135-7-1895 2482335

[35] De La Paz H Cooke SJ Heckels JE. Effect of sialylation of lipopolysaccharide of *Neisseria gonorrhoeae* on recognition and complement-mediated killing by monoclonal antibodies directed against different outer-membrane antigens. Microbiology (1995) 141:913–20. 10.1099/13500872-141-4-913 7539687

[36] Rice PA McQuillen DP Gulati S Jani DB Wetzler LM Blake MS. Serum resistance of *Neisseria gonorrhoeae*. Does it thwart the inflammatory response and facilitate the transmission of infection? Ann N Y Acad Sci. (1994) 730:7–14. 10.1111/j.1749-6632.1994.tb44234.x 8080215

[37] Gulati S Zheng B Reed GW Su X Cox AD St Michael F. Immunization against a saccharide epitope accelerates clearance of experimental gonococcal infection. PLoS Pathog. (2013) 9:e1003559. 10.1371/journal.ppat.1003559 24009500 PMC3757034

[38] Simpson SD Ho Y Rice PA Wetzler LM. T lymphocyte response to *Neisseria gonorrhoeae* porin in individuals with mucosal gonococcal infections. J Infect Dis. (1999) 180:762–73. 10.1086/314969 10438365

[39] Tsirpouchtsidis A Hurwitz R Brinkmann V Meyer TF Haas G. Neisserial immunoglobulin A1 protease induces specific T-cell responses in humans. Infect Immun. (2002) 70:335–44. 10.1128/IAI.70.1.335-344.2002 11748199 PMC127630

[40] Kraus SJ Perkins GH Geller RC. Lymphocyte transformation in repeated gonococcal urethritis. Infect Immun. (1970) 2:655–8. 16557891 10.1128/iai.2.5.655-658.1970 PMC416064

[41] Wyle FA Rowlett C Blumenthal T. Cell-mediated immune response in gonococcal infections. Br J Vener Dis. (1977) 53:353–9. 10.1136/sti.53.6.353 414816 PMC1045442

[42] Rarick M Mcpheeters C Bright S Navis A Skefos J Sebastiani P. Evidence for cross-regulated cytokine response in human peripheral blood mononuclear cells exposed to whole gonococcal bacteria *in vitro*. Microb Pathog. (2006) 40:261–70. 10.1016/j.micpath.2006.02.003 16626926

[43] Esquenazi V Streitfeld MM. Transformation of lymphocytes in gonorrhea before and after therapy. Infect Immun. (1973) 8:503–9. 4200538 10.1128/iai.8.4.503-509.1973 PMC422883

[44] Jain R Sonkar SC Chaudhry U Bala M Saluja D. In-silico Hierarchical approach for the identification of potential universal vaccine candidates (PUVCs) from *Neisseria gonorrhoeae*. J Theor Biol. (2016) 410:36–43. 10.1016/j.jtbi.2016.09.004 27596531

[45] Gagliardi MC Starnino S Teloni R Mariotti S Dal Conte I Di Carlo A. Circulating levels of interleukin-17A and interleukin-23 are increased in patients with gonococcal infection. FEMS Immunol Med Microbiol. (2011) 61:129–32. 10.1111/j.1574-695X.2010.00759.x 21214637

[46] Masson L Salkinder AL Olivier AJ Mckinnon LR Gamieldien H Mlisana K. Relationship between female genital tract infections, mucosal interleukin-17 production and local T helper type 17 cells. Immunology (2015) 146:557–67. 10.1111/imm.12527 26302175 PMC4693890

[47] Masson L Mlisana K Little F Werner L Mkhize NN Ronacher K. Defining genital tract cytokine signatures of sexually transmitted infections and bacterial vaginosis in women at high risk of HIV infection: a cross-sectional study. Sex Transm Infect. (2014) 90:580–7. 10.1136/sextrans-2014-051601 25107710

[48] Kaul R Rowland-Jones SL Gillespie G Kimani J Dong T Kiama P. Gonococcal cervicitis is associated with reduced systemic CD8+ T cell responses in human immunodeficiency virus type 1-infected and exposed, uninfected sex workers. J Infect Dis. (2002) 185:1525–9. 10.1086/340214 11992292

[49] Sheung A Rebbapragada A Shin LY Dobson-Belaire W Kimani J Ngugi E. Mucosal *Neisseria gonorrhoeae* coinfection during HIV acquisition is associated with enhanced systemic HIV-specific CD8 T-cell responses. AIDS (2008) 22:1729–37. 10.1097/QAD.0b013e32830baf5e 18753933

[50] Vieira VA Avelino-Silva VI Cerqueira NB Costa DA Costa PR Vasconcelos RP. Asymptomatic anorectal *Chlamydia trachomatis* and *Neisseria gonorrhoeae* infections are associated with systemic CD8+ T-cell activation. AIDS (2017) 31:2069–76. 10.1097/QAD.0000000000001580 28692536

[51] Liu Y Islam EA Jarvis GA Gray-Owen SD Russell MW. *Neisseria gonorrhoeae* selectively suppresses the development of Th1 and Th2 cells, and enhances Th17 cell responses, through TGF-beta-dependent mechanisms. Mucosal Immunol. (2012) 5:320–31. 10.1038/mi.2012.12 22354319 PMC3328619

[52] Witt K Veale DR Smith H. Resistance of *Neisseria gonorrhoeae* to ingestion and digestion by phagocytes of human buffy coat. J Med Microbiol. (1976) 9:1–12. 10.1099/00222615-9-1-1 817027

[53] Criss AK Katz BZ Seifert HS. Resistance of *Neisseria gonorrhoeae* to non-oxidative killing by adherent human polymorphonuclear leucocytes. Cell Microbiol. (2009) 11:1074–87. 10.1111/j.1462-5822.2009.01308.x 19290914 PMC2771623

[54] Liu Y Liu W Russell MW. Suppression of host adaptive immune responses by *Neisseria gonorrhoeae*: role of interleukin 10 and type 1 regulatory T cells. Mucosal Immunol. (2014) 7:165–76. 10.1038/mi.2013.36 23757303 PMC3812424

[55] Liu Y Egilmez NK Russell MW. Enhancement of adaptive immunity to *Neisseria gonorrhoeae* by local intravaginal administration of microencapsulated interleukin 12. J Infect Dis. (2013) 208:1821–9. 10.1093/infdis/jit354 24048962 PMC3814831

[56] Tapsall JW. Antibiotic resistance in *Neisseria gonorrhoeae*. Clin Infect Dis. (2005) 41 (Suppl 4):S263–8. 10.1086/430787 16032562

[57] Boslego JW Tramont EC Chung RC Mcchesney DG Ciak J Sadoff JC. Efficacy trial of a parenteral gonococcal pilus vaccine in men. Vaccine (1991) 9:154–62. 10.1016/0264-410X(91)90147-X 1675029

[58] Naess LM Aarvak T Aase A Oftung F Hoiby EA Sandin R. Human IgG subclass responses in relation to serum bactericidal and opsonic activities after immunization with three doses of the Norwegian serogroup B meningococcal outer membrane vesicle vaccine. Vaccine (1999) 17:754–64. 10.1016/S0264-410X(98)00259-X 10067680

[59] De Moraes JC Perkins BA Camargo MC Hidalgo NT Barbosa HA Sacchi CT. Protective efficacy of a serogroup B meningococcal vaccine in Sao Paulo, Brazil. Lancet (1992) 340:1074–8. 10.1016/0140-6736(92)93086-3 1357461

[60] Whelan J Klovstad H Haugen IL Holle MR Storsaeter J. Ecologic study of meningococcal B vaccine and *Neisseria gonorrhoeae* infection, Norway. Emerg Infect Dis. (2016) 22:1137–9. 10.3201/eid2206.151093 27191543 PMC4880101

[61] Petousis-Harris H Paynter J Morgan J Saxton P Mcardle B Goodyear-Smith F. Effectiveness of a group B outer membrane vesicle meningococcal vaccine against gonorrhoea in New Zealand: a retrospective case-control study. Lancet (2017) 390:1603–10. 10.1016/S0140-6736(17)31449-6 28705462

